# Efficient Particle Capture and Release Method for DNA Library Preparation on Microfluidics

**DOI:** 10.3390/mi16030332

**Published:** 2025-03-13

**Authors:** Zihan Song, Yihui Wu, Fengfeng Shu, Xiao Lv, Junyu Dong, Huan Li

**Affiliations:** 1 Changchun Institute of Optics, Fine Mechanics and Physics, Chinese Academy of Sciences, Changchun 130033, China; songzihan22@mails.ucas.ac.cn (Z.S.); lvxiao@ciomp.ac.cn (X.L.); 19330878716@163.com (J.D.); lihuan@ciomp.ac.cn (H.L.); 2 University of Chinese Academy of Sciences, Beijing 100049, China; 3 State Key Laboratory of Advanced Manufacturing for Optical Systems, Changchun 130033, China

**Keywords:** microfluidic, particle separation, particle release, synergistic effect, hydrodynamics

## Abstract

To address the issues of agglomeration during magnetic particle capture and the incomplete release of these particles during reuse in microfluidic chips for library preparation, a microchamber was utilized to enhance the dispersion area for magnetic particle capture. Additionally, the release of magnetic particles was achieved through the synergistic action of flow field and magnetic field. The simulation results indicated that as the inlet flow velocity varied from 0.02 m/s to 0.16 m/s and the magnet spacing ranged from 1.2 mm to 1.8 mm, the coverage of magnetic particles in the microchamber increased from 17.29% to 63.59%. Meanwhile, the magnetic particle capture rate decreased from 100% to 35.2%. These processes were further validated through experimental methods. During the release process, the trajectory of magnetic particles under the synergistic effect of flow field and magnetic field aligned with expectations. The captured magnetic particles were released from the microchamber within 12 s, achieving a release rate of 100%.

## 1. Introduction

Next-generation sequencing (NGS) has transformed molecular biology research by enabling the analysis of genomic, transcriptomic, and epigenomic features across a wide range of applications [1,2,3]. However, targeted NGS workflows often involve lengthy processing times and significant hands-on effort. Currently, the library preparation process, which includes target enrichment, remains the most labor-intensive part of the workflow. To prepare the completed library, it is necessary to isolate the target product from impurities. The most commonly used method relies on nucleic acid extraction using magnetic particles. These magnetic particles are surface-modified iron tetraoxide particles. Due to the superparamagnetism of iron tetraoxide, the magnetic particles only exhibit magnetism when influenced by an external magnetic field. This property provides them with excellent maneuverability, allowing for the effective control of the magnetic field and enabling the particles to be separated without the need for centrifugation [4,5].

Microfluidics is an emerging interdisciplinary field that combines physics, chemistry, biology, and materials science. It has significant applications in human genome research, clinical testing for infectious diseases, and the synthesis of novel drugs [6,7,8]. This technology is designed to handle and manipulate tiny volumes of fluid within microfluidic channels, which can be as small as micrometers or even nanometers. Methods for particle manipulation in microfluidics include acoustic [9,10,11,12], optical [13,14,15], and magnetic techniques [16,17,18]. Acoustic manipulation utilizes acoustic radiation forces generated by the transfer of momentum and energy between acoustic waves and particles, allowing for the non-contact handling of particles. This approach enables the precise control and gentle manipulation of various biological objects while maintaining good biocompatibility. Acoustic energy-induced drag has a large dependence on particle size, necessitating an increase in acoustic power to generate larger forces. However, nonlinear perturbations, such as acoustic flow, can occur and disrupt the trapping of these particles. Although optical tweezers provide versatility for trapping and releasing particles [19], they are limited to small particles and have low throughput, making them unsuitable for high-throughput continuous operation. Additionally, the optical equipment used in this technique tends to be complex and costly. Focused lasers generate very high energy levels that can alter the behavior of sensitive biological particles and can even damage biological samples. Magnetic particle traps do not involve physical contact and particles can be captured for long periods of time with little or no loss or degradation, this makes magnetic traps well suited for a wide range of applications, However, one disadvantage of magnetic traps is that the electromagnetic field generates a significant amount of heat over time [20,21,22,23]. A more commonly used method is to employ an external permanent magnet to generate the magnetic field. While this approach results in the quicker decay of magnetic field strength and lower resolution of magnetic bead control, it is much more direct and effective [24,25]. Additionally, a single magnetophoresis technique may struggle to handle various complex particle manipulation requirements. Therefore, combining multiple control methods may offer a more effective solution.

There have been various methods for separating magnetic particles using magnetic fields; however, these techniques still present some drawbacks. In microfluidic and microtubular channels at the micrometer scale, the high-throughput particle separation process tends to result in the formation of large aggregates due to significant dipole–dipole interactions [26]. Not only will the targeted analytes carried by the magnetic particles be trapped but other impurities will also be physically trapped within these aggregates. While the mechanism of particle capture has been extensively studied, the release and recovery of magnetic particles have often been overlooked in research [27]. In classical hydrodynamic theory, the flow rate of a uniformly flowing fluid in a microchannel approaches zero at the channel walls due to the effects of viscosity. This makes it challenging for the flow field to effectively influence the captured magnetic particles and resuspend them within the reagent. Most current research on releasing magnetic particles employs an active micromixer approach. However, all these methods necessitate the integration of external active devices, control circuits, and power supplies onto the microfluidic chip. This integration can be costly and complicated, increasing the risk of potential sample damage. Furthermore, treating the separation and release processes of magnetic particles in microfluidic chips as distinct areas of research significantly complicates system integration.

The microchamber offers a larger separation space for capturing magnetic particles, and the complex vortex environment enhances the dispersion of these particles, preventing their agglomeration. This improvement contributes to better overall product quality. By modifying the inlet flow channel, we can effectively control the trajectories of particles within the vortex environment. Additionally, the magnetic field provides a straightforward method for manipulating magnetic particles. It can be utilized not only for capturing these particles but also for altering their stable state, accelerating their movement in the flow field, and enabling their rapid release. This paper investigates the methods for capturing and rapidly releasing magnetic particles during library preparation. We employ numerical simulations to analyze the capture and release characteristics of magnetic particles in microchambers influenced by a magnetic field. We examine how variations in inlet flow rate and magnetic field distribution impact the capture rate and dispersion of magnetic particles. Furthermore, we explore the release mechanisms of magnetic particles under the synergistic effects of both the flow field and magnetic field, validating the simulation results with experimental methods. The integration of magnetic particle capture and release methods allows microfluidic-based nucleic acid extraction to operate without reliance on multiple active devices. This ensures compatibility and integration within microfluidic systems and significantly enhances the application of microfluidic technology in analytics, diagnostics, drug delivery, and other fields.

## 2. Model Construction

The motion of the magnetic particle in the microfluidic chip is mainly subject to the combined effect of the trailing force *F_d_* of the flow field, the magnetic field force *F_m_* of the gradient magnetic field, the gravity force *F_g_*, and the buoyancy force *F_f_*. It is known based on Newton’s second law:

(1) $$m_{P}\frac{du_{P}}{dt}=F_{m}+F_{d}+F_{g}+F_{f},$$

where *u_p_* is the particle velocity and *m_p_* is the particle mass. The motion of the magnetic particles in the microfluidic channel can be regarded as the motion of regular spherical particles in a laminar flow, and the trailing force received by the particles in the flow field should be calculated using Stokes’ law:

(2) $$F_{d}=6\pi \eta R_{P}\left(u-u_{P}\right),$$

where η, *R_P_*, and *u* are the medium viscosity, particle radius, and medium velocity. The magnetic field force on a magnetic particle in a microfluidic system subjected to a gradient magnetic field can be calculated using the magnetic dipole moment method, which calculates the magnetic field force on the particle in a gradient magnetic field by equating the magnetic bead particle to a point magnetic dipole:

(3) $$F_{m}=\nabla \left(m\cdot B\right);$$

the magnetic moment of a magnetic particle can be expressed as $m=V_{P}\chi_{b}H$, where *V_p_* is the particle volume and $\chi_{b}$ is the magnetic susceptibility of the particle. The combined force of gravity and buoyancy is three orders of magnitude smaller than drag and magnetic field forces, so the influence of gravity and buoyancy is ignored.

**Figure 1:**
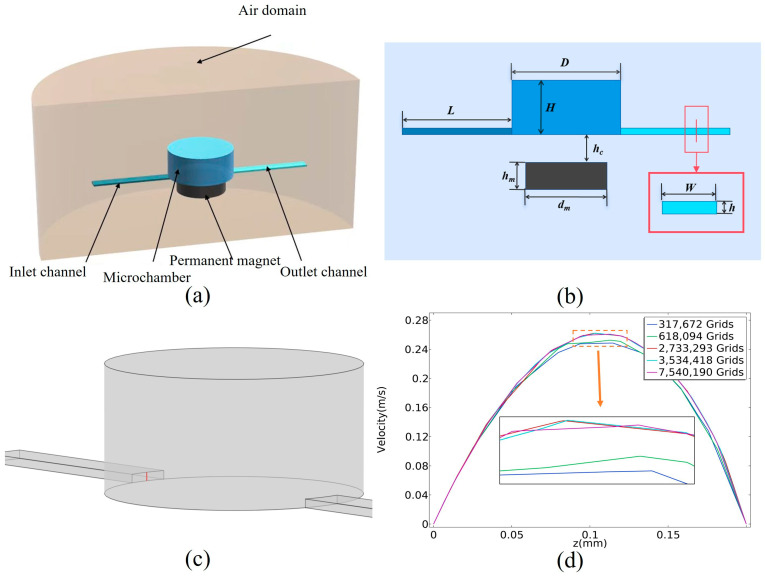
Numerical simulation simplified model. (**a**) Three-dimensional model of computational domain. (**b**) Plane structure of computational model. (**c**) Longitudinal velocity magnitude distribution on specified line. (**d**) Mesh independency analysis of line.

Using a three-dimensional finite element software COMSOL 6.0 model to solve the governing partial differential equations, the model of this paper uses the COMSOL physics field laminar flow module magnetic field (no current), and particle tracking module. Figure 1a,b show the simplified model for the magnetic particle capture motion characterization study. The model is divided into five computational domains, with flow field and particle calculations in the inlet channel, cavity, and outlet channel regions, and magnetic field calculations in all regions. The flow distribution of the fluid is obtained by solving the Navier–Stokes equation, and the flow dynamics boundary is no-slip. The boundary condition of the outlet is set to pressure (the static pressure is 0 Pa). The boundary of the air domain is set to zero standard magnetic potential, with a radius of 20 mm and a height of 24 mm. A tetrahedral mesh is used for the three-dimensional model. The pseudo time step for the transient solver is 0.00001 s. Considering that the library mixing solution after preparation is about 50 μL, and the amount of magnetic particles solution is 30 μL, the volume of the cylindrical microchamber for magnetic particle processing should be more than 80 μL, so the diameter of the microchamber is D = 6 mm and the height is H = 3 mm. The inlet is located on the left side of the chamber, with a width and a height of w = 1 mm and h = 0.2 mm, The fluid needs to be fully stabilized after entering the microchannel, and therefore the channel length can be chosen as long as 3 mm. The magnetic source utilized is a NdFeB magnet with a diameter and height of d_m_ = 5 mm and h_m_ = 1.5 mm. This magnet is chosen because of its small size and high magnetic field strength and gradient. And h_c_ indicates the distance between the magnet and the microchamber, with h_c_ = 1.5 mm for initial conditions. The outlet is located on the right side of the microchamber and has the same parameters as the inlet.

The density of magnetic particles is defined as ρ_p_ = 1.4 × 10^3^ kg/m^3^, the particle size as d_p_ = 4 μm, and the particle relative permeability as u_rp_ = 1000. The motion of the particles within the microfluidic chip is primarily influenced by the combined effects of the trailing force, the magnetic field force from the gradient magnetic field, gravity, and buoyancy. The calculations indicate that the combined forces of gravity and buoyancy acting on the magnetic particles in the fluid are three orders of magnitude smaller than the trailing and magnetic forces, allowing their effects on the calculations to be ignored. When the magnetic particles are moved by the fluid’s trailing force, they exert a reaction force on the fluid. Due to the small size of the magnetic particles relative to the dimensions of the microfluidic channel and the low concentration of the magnetic particle solution used, these particles do not possess enough inertia to significantly perturb the fluid. In modeling the system under these conditions, the motion of the particles is unidirectionally coupled with both the flow field and the magnetic field. Consequently, the distributions of the flow and magnetic fields can be computed, and the trajectories of the particles over time can be determined using the equations of motion for the magnetic particles. The velocity distribution error of the cutoff is calculated by solving the velocity distribution of the longitudinal flow field at the inlet of the cavity under different grid divisions. As shown in Figure 1c,d, when using the 2,733,293 grid, the velocity distribution of the intercept is approximately the same as using the 7,540,190 grid, so the 2,733,293 grid is used for calculation.

## 3. Research on Separation Characteristics of Magnetic Particles

### 3.1. Effect of Flow Field on Magnetic Particle Capture

Due to the chamber’s volume requirements, its depth is significantly greater than that of the microchannel. As a result, the Reynolds number cannot be maintained within the laminar flow range, leading to a complex flow field that contains vortices. Before studying the impact of the flow field on the particles, it is necessary to investigate how different inlet flow velocities affect the flow field within the chamber.

**Figure 2:**
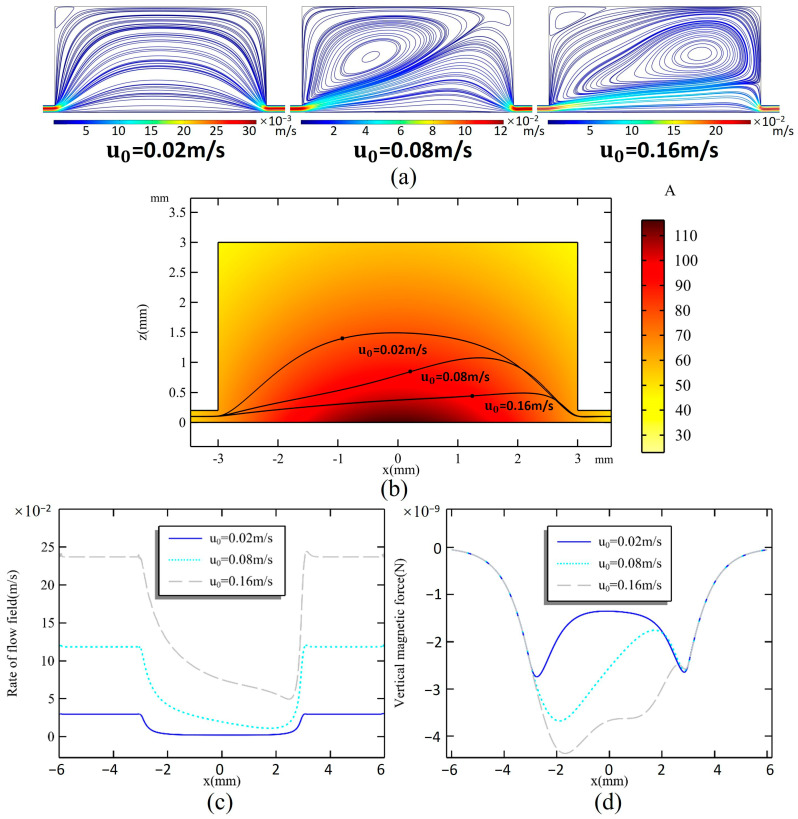
(**a**) Flow profiles of the microchamber at inlet flow rates u_0_ of 0.02 m/s, 0.08 m/s, and 0.16 m/s. (**b**) Magnetic field distribution in the microchamber and trajectory of the center streamline when u_0_ = 0.02, 0.08, and 0.16 m/s. (**c**) Rate changes on the streamline. (**d**) The simulated magnetic force on a magnetic particle when moving along the streamline.

Figure 2a illustrates the relationship between different inlet flow rates and the distribution of the flow field within the microchamber. It is clear that the volume occupied by the vortex flow field in the microchamber increases as the inlet flow rate rises. At an inlet flow rate of u_0_ = 0.02 m/s, the vortex flow field does not impact the overall distribution; the streamlines enter the microchamber from the inlet and quickly fill the entire chamber. However, at u_0_ = 0.08 m/s, a significant vortex field is generated at the inlet, causing the trajectory of the streamlines to deform due to the influence of the vortex field. As the flow rate increases beyond u_0_ = 0.16 m/s, the vortex field entirely occupies the upper section of the chamber, while the streamlines tend to remain close to the bottom of the chamber after entering from the inlet. The trajectory and flow rate changing of the center streamline are shown in Figure 2b,c; with the increase in the inlet flow rate, the flow rate in the microchannel increases linearly. The streamline begins to expand due to space enlargement since it enters the microchamber; at this time, the flow field rate along the streamline decreases rapidly. As the flow field approaches the inlet, the trajectory begins to contract, and the flow rate increases rapidly and reaches the same as the inlet in a short time.

The shape of the streamline influences the trajectory of magnetic particles within the flow field. This is due to the fact that the magnetic field generated by the permanent magnet is not uniform, resulting in varying sizes and directions of the magnetic force acting on the particles as they move along different paths. The component of the magnetic force that is perpendicular to the streamline is particularly significant in capturing the particles. Figure 2d illustrates the vertical magnetic force exerted on a magnetic particle as it travels along a streamline. The magnetic force acting on particles exhibits significant variation depending on their trajectory. However, within the microchannel, there is no difference in magnetic force for particles following the same path. Upon entering the chamber, the streamlines expand to varying degrees, resulting in peak magnetic forces at different locations that decline rapidly. At a flow rate of 0.02 m/s, the magnetic force is strongest at the edges of the microchamber, measuring a maximum of 2.74 × 10^−9^ N, while it is weaker in the center. When the flow rate increases to 0.08 m/s, the magnetic force in the front half of the microchamber rises. As the flow rate continues to increase to 0.16 m/s, the magnetic force in the center of the microchamber becomes stronger than that at the edges.

**Figure 3:**
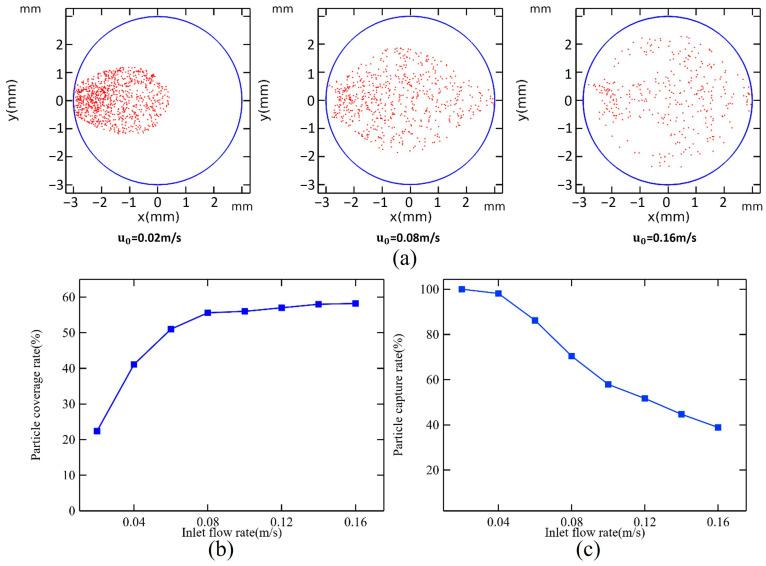
(**a**) Magnetic bead distribution at flow rates of 0.02 m/s, 0.08 m/s, and 0.16 m/s. Effect of u_0_ on (**b**) particle coverage rate and (**c**) particle capture rate when h_c_ = 1.5 mm.

The process of capturing magnetic particles is simulated for flow rates ranging from 0.02 m/s to 0.16 m/s. Figure 3a illustrates the distribution of 1000 magnetic particles released from the inlet. Figure 3b,c show the particle coverage rate, which reflects the area covered by magnetic particles relative to the total area of the microchamber’s bottom, and can be obtained as follows:

(4) $$COR=\frac{Particles\;coverage\;area\;(S_{p})}{Chamber\;bottom\;area\;(S)}\times 100\%;$$

and the particle capture rate, which indicates the percentage of captured magnetic particles compared to those released:

(5) $$CPR=\frac{Captured\;particles\;number\;(N_{c})}{Particles\;total\;number\;(N)}\times 100\%.$$

At a flow rate of 0.02 m/s, all magnetic particles are captured; however, they are primarily concentrated in the first half of the microchamber, covering only 22.4% of the chamber’s area. As the inlet flow rate increases, the magnetic particle capture rate starts to decline. When the flow rate reaches 0.16 m/s, the capture rate stabilizes at only 38.9%. Increasing the flow rate within a certain range positively influences magnetic particle coverage. It peaks at 55.6% when the flow rate is 0.08 m/s, after which further increases to the flow rate do not significantly affect coverage. At a flow rate of 0.16 m/s, the trailing force becomes the dominant factor influencing the movement of magnetic particles, resulting in a distribution that mirrors the flow field’s shape.

### 3.2. Effect of Magnetic Field on Magnetic Particle Capture

**Figure 4:**
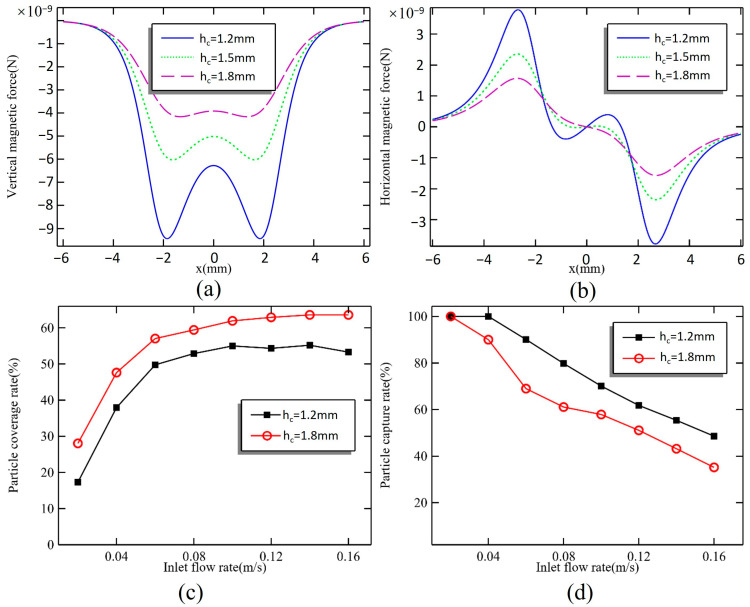
(**a**) Vertical magnetic force and (**b**) horizontal magnetic force on the bottom surface when h_c_ = 1.2, 1.5, and 1.8 mm. Effect of u_0_ on (**c**) particle coverage rate and (**d**) particle capture rate when h_c_ = 1.2 and 1.8 mm.

The magnetic field produced by permanent magnets is known to decay quickly. A high magnetic field gradient generates a stronger magnetic force, which can increase the capacity to capture magnetic particles. However, this also tends to centralize the magnetic particles at the location of the stronger magnetic force, affecting the uniform distribution of the particles. When the h_c_ is set at 1.2, 1.5, and 1.8 mm, the distribution of magnetic force on the bottom surface of the microchamber is illustrated in Figure 4a,b.

In the perpendicular direction, when h_c_ = 1.2 mm, the peak magnetic force reaches 9.44 × 10^−9^ N, while at the center of the microchamber, the magnetic force is only 6.28 × 10^−9^ N. When h_c_ is increased to 1.5 mm, the peak magnetic force decreases to 6.03 × 10^−9^ N, representing a 36.2% reduction. In contrast, the magnetic force at the center only decreases by 20%. At a distance of 1.8 mm, there are no significant sharp peaks in the magnetic force on the bottom surface of the microchamber; the maximum magnetic force recorded is 4.16 × 10^−9^ N, with a difference of only 0.24 × 10^−9^ N compared to the force at the center. As the spacing between the magnet and microchamber increases, the overall magnetic force declines. The rate of reduction for the peak magnetic force is greater than that for the force at the center, leading to a more homogenized magnetic field distribution. The magnetic force in the horizontal direction plays a critical role in the distribution of magnetic particles, directing them firmly toward the center of the microchamber. When the distance between the magnet and the microchamber is 1.2 mm, there is a magnetic force acting toward the circumference of a small area at the chamber’s center. However, this force is significantly lower than the maximum value of 3.78 × 10^−9^ N, and its influence is limited, making it negligible. At distances of 1.5 mm and 1.8 mm, the maximum magnetic force decreases to 2.36 × 10^−9^ N and 1.57 × 10^−9^ N, marking a reduction of 37.6% and 58.5%, respectively.

Figure 4c,d illustrate the trend of magnetic particle capture results for varying inlet flow rates from 0.02 m/s to 0.16 m/s at different magnet spacings. Reducing the magnetic force effectively increases the coverage area of the magnetic particles; however, it also leads to a higher number of lost particles. The ability to capture magnetic particles improves when the height of the magnet is set to h_c_ = 1.2 mm, and there is no loss of particles when the flow rate is 0.04 m/s. At this flow rate, magnetic particles are captured earlier due to the stronger peripheral magnetic force, resulting in a more concentrated distribution. As the flow rate exceeds 0.1 m/s, the magnetic particle coverage gradually stabilizes at around 55%. When the magnet height is increased to 1.8 mm, the coverage reaches 64% at higher flow rates, demonstrating that adjusting the magnet spacing can enhance the dispersion of magnetic particles.

### 3.3. Experimental Verification of Magnetic Particle Capture

**Figure 5:**
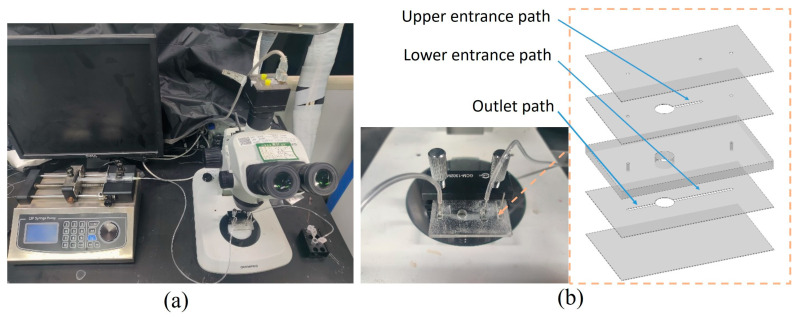
Microfluidic experiment bench. (**a**) Experimental equipment. (**b**) Microfluidic chip.

The experimental platform based on the research content is illustrated in Figure 5. The microfluidic chip was fabricated using the Poly(methyl methacrylate)(PMMA) engraving method. The microchannel layer, chamber layer, and cover plate were processed with a laser engraver and then laminated together with 3M adhesive. A steel needle and base were fixed to the microfluidic chip using a light-curing adhesive, completing the assembly of the chip. To prepare the magnetic particle solution, we diluted a 2.5% original magnetic particle solution with deionized water in a ratio of 1:50. The magnetic bead solution was placed in a syringe, and a high-precision syringe pump controled the injection speed, pushing the magnetic beads into the chip while discharging the waste liquid from the outlet and collecting it in a reagent tube. A magnet with a diameter of 5 mm and a thickness of 1.5 mm, graded N35, was positioned directly beneath the chamber. The syringe pump controlled the rate from 4 μL/s to 32 μL/s. The distribution of captured magnetic particles was observed under a microscope, and the experimental results were recorded using a high-speed camera.

**Figure 6:**
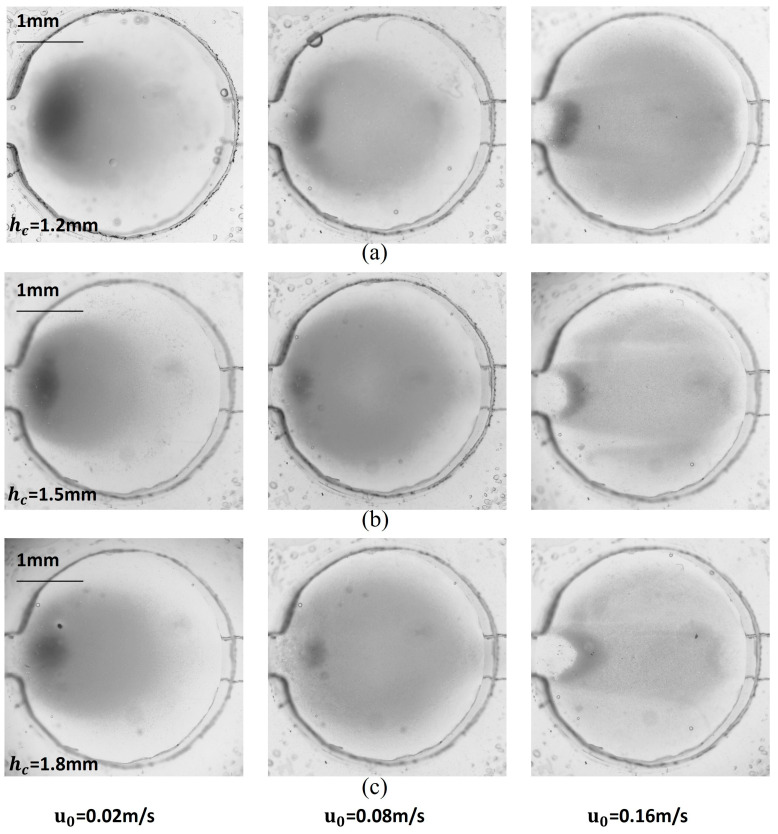
Image of capture results for magnetic particle solution with 0.05% mass fraction when h_c_ = (**a**) 1.2 mm, (**b**) 1.5 mm, and (**c**) 1.8 mm.

The distribution of magnetic particles in the microchamber under various conditions is illustrated in Figure 6. The distribution at the bottom of the microchamber generally aligned with the simulation results, though there were slight differences in shape, with actual magnetic particle coverage being better than what was simulated. At low flow rates, the magnetic force significantly influenced the magnetic particles more than the trailing force. Consequently, the particles tended to accumulate primarily in the front half of the microchamber, particularly heavy at the inlet. In contrast, at high flow rates, the distribution of magnetic particles revealed clear flow field characteristics. The area covered by the particles increased and occupied most of the bottom surface, yet the total number of captured magnetic particles decreased significantly. The spacing between the magnet and the microchamber also impacted particle distribution. When the spacing was small, the magnetic particles were more strongly affected by the magnetic field at the edge of the magnet, leading to a concentration in the annular region, while fewer particles were found in the center. Conversely, increasing the space between the magnet and the microchamber reduced the lateral magnetic force on the particles, resulting in a more uniform distribution. However, this increased spacing diminished the magnetic field’s effectiveness in holding the particles, leading to a higher loss of magnetic particles at elevated flow rates.

According to the distribution image of magnetic particles obtained from the experiment, it is impossible to calculate the number of particles directly. Therefore, to analyze the separation characteristics of particles, this section refers to the number of magnetic particles using the grayscale value of the chamber part image. When calculating the grayscale value, a grayscale threshold needs to be set to exclude the influence of background grayscale. If the grayscale threshold is set as the average value of the background grayscale, the coverage of magnetic particles can be expressed as:

(6) $$COR=\frac{Partice\;pixels\;number\;(P_{p})}{Chamber\;pixels\;number\;(P)}\times 100\%.$$

The particle number can be expressed as $I=\sum_{(i,j)\epsilon S}\left(G_{ij}-G\right)$, *G_ij_* is the grayscale value of each pixel in the chamber, and *G* is the average grayscale value of the chamber background. Setting the number of magnetic particles with the rate of 0.02 m/s as the initial number *I*_1_, the particle capture rate can be expressed as:

(7) $$CPR=\frac{Captured\;particles\;number\;(I_{c})}{Initial\;particles\;number\;(I_{1})}\times 100\%.$$

**Figure 7:**
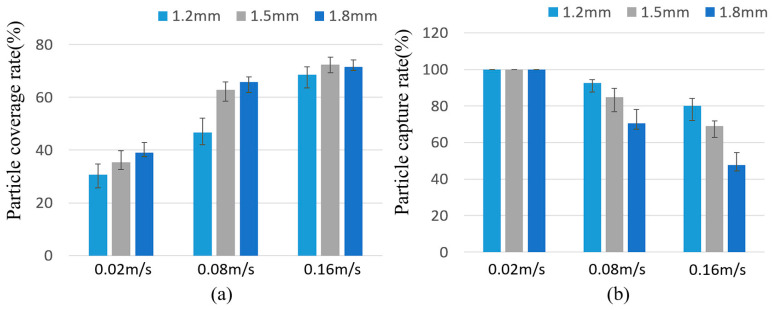
(**a**) Particle coverage rate and (**b**) particle capture rate when h_c_ = 1.2, 1.5, and 1.8 mm.

Based on this, the separation characteristics of the magnetic particles were calculated as shown in Figure 7. It can be seen that the separation characteristics of the magnetic particles measured in the experiment were consistent with the simulation results as a whole. There was no significant difference in the coverage of the particles at the rate of 0.16 m/s. This is because some particles were captured at the bottom of the cavity and were subjected to a horizontal magnetic field force that overcame the frictional force between the magnetic particles and the chamber surface, resulting in a decrease in both coverage and capture rates.

## 4. Research on the Release Process of Magnetic Particles

The prolonged capture of magnetic particles increases the likelihood of particle agglomeration and irreversible adsorption. This not only reduces nucleic acid extraction yields but also affects the reusability of the microfluidic system. In the process of nucleic acid extraction using magnetic particles, the washed particles must be released and resuspended in a buffer or other reagents. Typically, the flow rate decreases significantly when the flow field enters the microchamber, especially near the wall, where it approaches zero due to viscous effects. As a result, it becomes challenging for the magnetic particles at the bottom of the microchamber to return to a levitated state solely due to the flow field. To facilitate this, a magnet can be placed above the microchamber, drawing the magnetic particles back into a levitated state after they leave the low flow rate region. Once the particles are elevated, the flow field can effectively influence them, enabling a rapid release of the magnetic particles and allowing for efficient subsequent processing. The motion characteristics of magnetic particle release, influenced by the combined effects of the vortex field and magnetic field under different conditions, were analyzed through simulation and experimental validation.

When the inlet is positioned at the bottom of the microchamber, the main flow field is primarily concentrated near the center axis. In this scenario, a vortex field develops at the bottom sides of the microchamber, which expands as the inlet flow rate increases. As a result, a higher inlet flow field can lead to a greater number of magnetic particles being trapped in the vortex field, which is detrimental to their release. The coexistence of the main flow field and the vortex field at the bottom of the microchamber makes the movement of magnetic particles uncontrollable. In contrast, the upper layer of the microchamber exhibits a consistent flow field direction. It is reasonable to suggest that if the inlet were relocated to the upper part of the microchamber, it would invert the flow field distribution. This change would position the magnetic particles in an area with a uniform flow direction, allowing for more effective control of the magnetic particles through the flow field.

**Figure 8:**
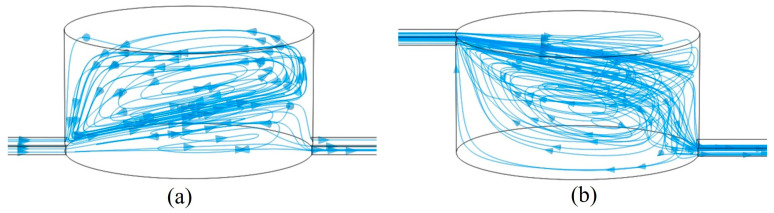
Streamline diagram in the cavity with (**a**) a lower path and (**b**) an upper path. The arrows indicate the direction of flow field motion.

Figure 8 illustrates the distribution of streamlines in the microchamber at different inlet positions. At high flow rates, when the inlet is positioned below the microchamber, the primary flow field occupies only a small area at the bottom of the chamber. Surrounding this main flow field is a vortex field that moves in the same direction as the main flow at the surface, but in the opposite direction at the boundary of the flow field. In this scenario, magnetic particles within the main flow field move rapidly toward the outlet, while those in the vortex field spiral upward and are eventually captured by the magnetic field. Conversely, when the inlet is located at the upper part of the microchamber, most of the lower surface is dominated by a vortex flow field that also moves in a clockwise direction along the walls, ultimately returning to the main flow field at the inlet. In this case, most magnetic particles are influenced by the vortex flow field, initially moving away from the exit and converging close to the wall before finally entering the exit microchannel along the lower surface of the main flow field.

### 4.1. Analysis of Factors Affecting Magnetic Bead Release

The upper magnet is positioned farther away from the captured magnetic particles, which means that the particles are less influenced by the magnetic field at the start of the release phase. To enhance the release process of the magnetic particles, increasing the strength of the magnetic field at the bottom of the microchamber is beneficial. As the magnetic particles move to the upper layer of the microchamber, the distance between them and the magnet decreases significantly, and excessive magnetic force can cause the particles to become trapped on the upper surface of the microchamber.

**Figure 9:**
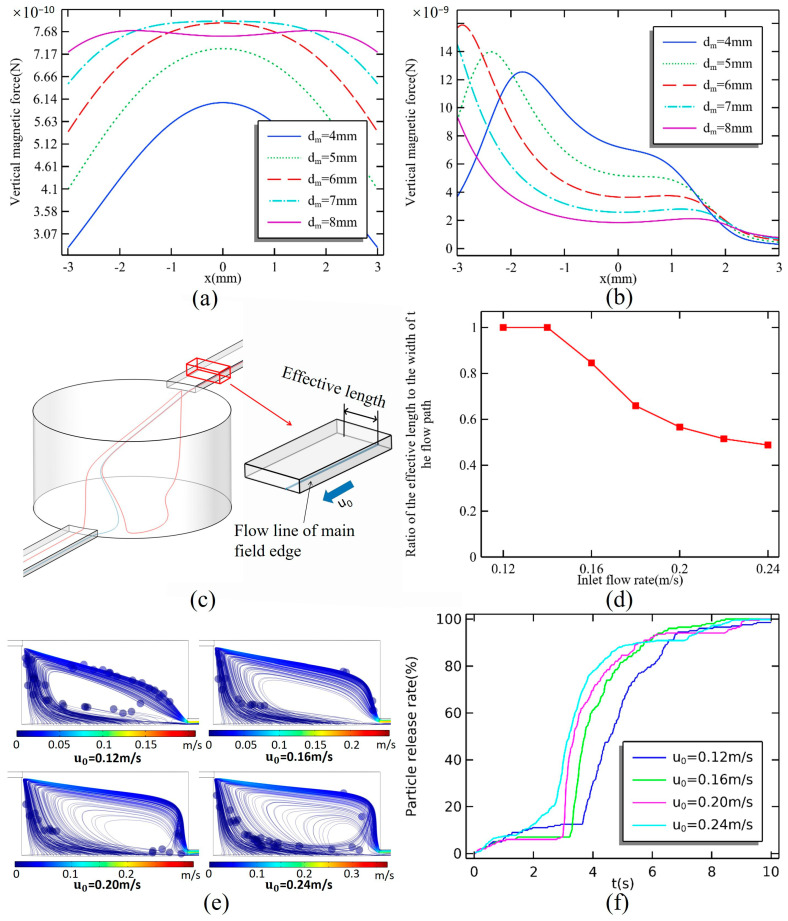
Magnetic force on (**a**) the bottom surface and (**b**) the center streamline. (**c**) Two streamlines with close starting positions but different patterns; the streamline (blue) closer to the center is the main field edge streamline, and the streamline (red) further from the center forms the vortex field. (**d**) Effect of flow rate on the ratio of the main flow field width (effective length) starting at the inlet bottom to the width of the flow path. (**e**) Instantaneous trajectory of magnetic particles after the stable operation of the system for 6 s. (**f**) Plot of flow rate versus particle release process.

Figure 9a,b illustrate the magnetic force on the lower surface of the microchamber and the flow field within the center of the main flow field from magnetic sources of varying diameters. As the diameter of the magnet increases, the magnetic force at the edge of the bottom surface also increases. The force at the center of the bottom surface reaches its maximum when the magnet’s diameter matches that of the microchamber, after which it begins to decrease with further increases in magnet diameter. At the edge of the flow field, the flow rate is relatively lower, resulting in less force on the magnetic particles. A larger magnetic force improves release efficiency. However, it is essential to minimize the magnetic force in the center flow field of the main flow field to prevent the magnetic particles from being captured by the magnetic field while moving through the upper layer of the microchamber. Consequently, the optimal diameter for the magnet is determined to be 8 mm.

The trajectories of magnetic particles are closely aligned with the streamlines that originate at the bottom of the inlet. However, not all streamlines that start at the inlet lead directly to the exit. The streamline that begins below the inlet side creates a complex flow trajectory. Magnetic particles entering this region will also circulate within the vortex field, following the path of the streamline. Since streamlines do not intersect, the streamline nearest to the vortex field also serves as the boundary streamline of the main flow field. The distance between the starting points of the left and right boundary streamline is referred to as the effective length of the inlet width. Figure 9c,d illustrate the relationship between effective length and inlet flow rate, with flow rates ranging from 0.02 m/s to 0.24 m/s. When the flow rate is less than 0.14 m/s, the effective length is equal to the inlet width. However, at a flow rate of 0.16 m/s, the effective length decreases to 84.6% of the inlet width, as some of the streamlines begin to enter the vortex field. As the inlet flow rate continues to increase, the effective length continues to diminish, although the rate of decrease slows with rising flow rates. At an inlet flow rate of 0.24 m/s, the effective length is only 48.8% of the inlet width.

Figure 9e,f illustrate the comparison of magnetic particle release profiles at inlet flow rates ranging from 0.12 m/s to 0.24 m/s. The particle release rate can be expressed as:

(8) $$RER=1-\frac{particles\;number\;in\;the\;chamber(N_{p})}{Particles\;total\;number(N)}\times 100\%.$$

The magnet has a diameter of 8 mm, and there is a spacing of 0.8 mm between the magnet and the microcavity. During the release process, magnetic particles located in the main flow field exit the microchamber more quickly than those in the vortex field, which initially experience a slow rise phase. The path taken by the magnetic particles in the vortex field is longer, causing a temporary stall in the growth of the release curve. At an inlet flow rate of 0.12 m/s, magnetic particles from the main flow field are released at a faster rate, accounting for 12.5% of the total amount. The release rate of magnetic particles is positively correlated with the inlet flow rate until t = 6 s. At this point, the curves for inlet flow rates of 0.16 m/s to 0.24 m/s converge at a single point. After t = 6 s, the relationship between release efficiency and inlet flow rate becomes negative. This shift is attributed to an increased number of magnetic particles becoming trapped in the vortex flow field, which reduces the overall release rate. Additionally, no return of magnetic particles to the vortex field occurs at flow rates of 0.12 m/s and 0.16 m/s.

### 4.2. Experimental Study on Magnetic Particle Capture

**Figure 10:**
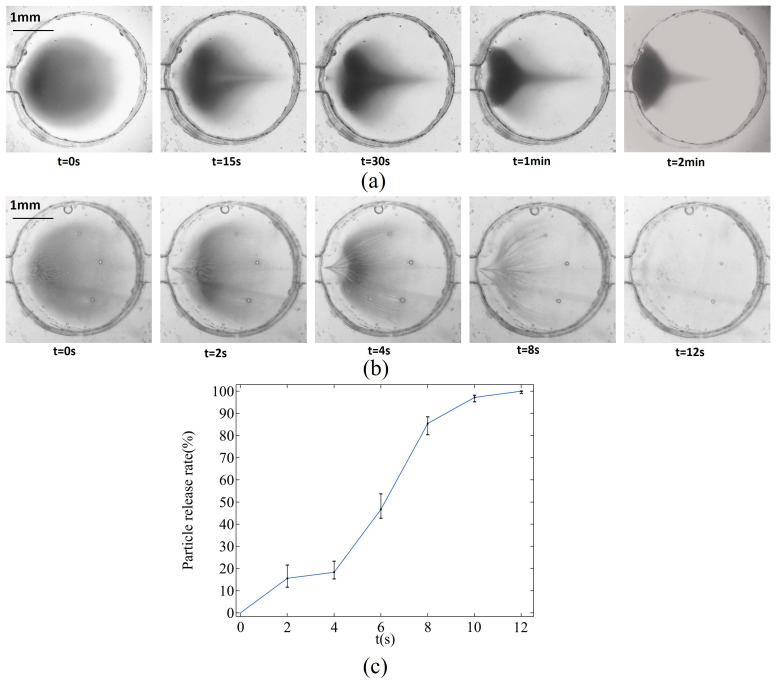
Movement of magnetic beads in the microchamber. (**a**) Without permanent magnets, the particles converge at the inlet but are deposited at the bottom of the microchamber and cannot be released. (**b**) Magnetic particles move faster in the presence of permanent magnets and are released from the microchamber along the flow field. (**c**) Particle release rate with magnet.

The particle release experiment utilizes the same equipment as the particle capture experiment. In this setup, particles are first captured at the bottom of the chamber. The inlet is then switched to the upper flow path while the lower flow path is closed, and the reagents are replaced with deionized water. After starting the syringe pump, the magnet positioned underneath the microfluidic chip is removed, and a new magnet is placed at a certain height directly above the chamber. The movement of the magnetic particles is observed and recorded at a flow rate of 0.16 m/s using a microscope and a high-speed camera. Additionally, a case without a permanent magnet is set up as a control. The status of magnetic particle motion at different time points is illustrated in Figure 10a,b.

Magnetic particles initially move slowly without magnetic assistance. At t = 15 s, the magnetic particles near the outlet on both sides begin to migrate toward the inlet due to the flow field’s influence. By t = 1 min, these particles continue to move in the inlet direction, while the particles along the central axis remain largely unaffected by the flow and do not move at this time. As the flow rate weakens at the edge of the flow field, there is not enough force to propel the magnetic particles upward. By t = 2 min, the particles become tightly packed against the chamber’s side walls, and their movement comes to a standstill. After introducing a magnet, the migration speed of the magnetic particles significantly increases, and their movement is no longer confined to the bottom surface of the chamber. The magnets pull the particles upward, and as they approach the magnets, the force of attraction becomes the dominant influence, causing the particles to move from the inlet to the outlet along the center axis. Grayscale processing is performed on the image and the release rate of particles is calculated as follows:

(9) $$RER=1-\frac{Particles\;number\;in\;the\;chamber\;(I_{t})}{Initial\;particles\;number(I_{0})}\times 100\%$$

In Figure 10c, the release rate of particles shows a slow–fast–slow trend, which is similar to the simulation curve to a certain extent. However, the experimental curve has a small change amplitude. This is because particles not only move in a plane in the cavity, but they also leave the microscope focal plane when they move upward, making it difficult to capture particle images.

## 5. Discussion and Conclusions

In this work, we demonstrate that magnetism can be utilized not only for capturing magnetic particles but also for facilitating their release. The chamber design offers an expanded space for particle dispersion and reduces the likelihood of impurities caused by bead aggregation. Continuous flow microfluidic systems can also assist in particle concentration and purification by employing a cycle of trapping and releasing. However, enhancing the washing effect often results in an increase in device size. The primary advantage of chamber microfluidics is its compact size, which can lead to issues with residual particles remaining in the chamber. Actively driven techniques typically offer better control and higher throughput; however, they may lack seamless integration with other platforms. The magnetically assisted hydrodynamic bead release method addresses this integration challenge. In our approach, a regular flow field is established between the upper inlet and the lower outlet, facilitating the release of magnetic beads within the cavity. During this process, particles are primarily influenced by both magnetic force and the boundary flow field at the lower layer of the inlet. An increase in magnetic force causes beads to detach more rapidly from the low-velocity flow field at the bottom. Additionally, the boundary flow field at the lower inlet determines the particles’ trajectories. As the flow velocity increases, the stable flow field region decreases. At the same time, a stronger magnetic force can cause magnetic particles to deviate from their intended flow paths and enter an unstable region, Moreover, this method holds the potential for rapid mixing in dual chambers.

This paper presents a method for controlling magnetic beads in microfluidic library preparation, enabling the separation and release of magnetic beads within the same reaction chamber. Numerical simulations are conducted to analyze how variations in inlet flow rates and magnet parameters affect the motion patterns of magnetic particles in the microchamber. The simulation results are validated through experimental methods. The design of the volumetric chamber increases the surface area for dispersing magnetic beads, effectively addressing the issue of bead over-concentration. Additionally, the synergistic effect of the magnetic field and flow field allows for the release of magnetic beads within the volumetric chamber without creating any dead zones. This approach facilitates the integration of particle capture and release technologies, ensuring compatibility and consistency within the microfluidic system.

## Data Availability

The original contributions presented in this study are included in the article. Further inquiries can be directed to the corresponding authors.
